# CpH methylome analysis in human cortical neurons identifies novel gene pathways and drug targets for opioid use disorder

**DOI:** 10.3389/fpsyt.2022.1078894

**Published:** 2023-01-19

**Authors:** Sheila T. Nagamatsu, Gregory Rompala, Yasmin L. Hurd, Diana L. Núñez-Rios, Janitza L. Montalvo-Ortiz, Traumatic Stress Brain Research Group, Victor E. Alvarez

**Affiliations:** ^1^Division of Human Genetics, Department of Psychiatry, Yale University School of Medicine, New Haven, CT, United States; ^2^VA Connecticut (VA CT) Healthcare Center, West Haven, CT, United States; ^3^Clinical Neurosciences Division, U.S. Department of Veterans Affairs National Center of Posttraumatic Stress Disorder, West Haven, CT, United States; ^4^Icahn School of Medicine at Mount Sinai, New York, NY, United States

**Keywords:** opioid, methylation, non-CpG site, postmortem human brain, orbitofrontal cortex, epigenetic

## Abstract

**Introduction:**

DNA methylation (DNAm), an epigenetic mechanism, has been associated with opioid use disorder (OUD) in preclinical and human studies. However, most of the studies have focused on DNAm at CpG sites. DNAm at non-CpG sites (mCpHs, where H indicates A, T, or C) has been recently shown to have a role in gene regulation and to be highly abundant in neurons. However, its role in OUD is unknown. This work aims to evaluate mCpHs in the human postmortem orbital frontal cortex (OFC) in the context of OUD.

**Methods:**

A total of 38 Postmortem OFC samples were obtained from the VA Brain Bank (OUD = 12; Control = 26). mCpHs were assessed using reduced representation oxidative bisulfite sequencing in neuronal nuclei. Differential analysis was performed using the “methylkit” R package. Age, ancestry, postmortem interval, PTSD, and smoking status were included as covariates. Significant mCpHs were set at *q*-value < 0.05. Gene Ontology (GO) and KEGG enrichment analyses were performed for the annotated genes of all differential mCpH loci using String, ShinyGO, and amiGO software. Further, all annotated genes were analyzed using the Drug gene interaction database (DGIdb).

**Results:**

A total of 2,352 differentially methylated genome-wide significant mCpHs were identified in OUD, mapping to 2,081 genes. GO analysis of genes with differential mCpH loci showed enrichment for nervous system development (*p*-value = 2.32E-19). KEGG enrichment analysis identified axon guidance and glutamatergic synapse (FDR 9E-4–2.1E-2). Drug interaction analysis found 3,420 interactions between the annotated genes and drugs, identifying interactions with 15 opioid-related drugs, including lofexidine and tizanidine, both previously used for the treatment of OUD-related symptoms.

**Conclusion:**

Our findings suggest a role of mCpHs for OUD in cortical neurons and reveal important biological pathways and drug targets associated with the disorder.

## Introduction

Opioid use disorder (OUD) is a chronic lifelong disorder at epidemic levels in the United States (1). OUD has been associated with a high disease burden and overdose death rates (17.8 per 100,000 individuals) (2). While genetic risk factors have been identified in recent large-scale genome-wide association studies (GWAS) of OUD (3, 4), these explain only part of the variance. Environmental factors interplay with the genetic background to influence OUD risk, possibly *via* epigenetic mechanisms. Alterations in DNA methylation (DNAm), one of the epigenetic mechanisms, have been associated with OUD. DNAm generally occurs in cytosine-guanine dinucleotides linked by a phosphate group (CpG site) and is associated with gene repression when located in the CpG island of the promoter region (5). Our group and others have identified differentially methylated CpG sites associated with OUD in studies examining peripheral tissue (6–8).

In the human postmortem brain, a previous study evaluating DNAm using the Infinium HumanMethylation450K BeadChip identified 1,298 differential 5mC in the human postmortem orbitofrontal cortex (OFC) of heroin users (*p* < 0.001) (9). The OFC is known to be involved in decision-making processes related to drug addiction (10–12). A recent study evaluating DNAm using the Illumina MethylationEPIC BeadChip (EPIC) array in the dorsolateral prefrontal cortex (dlPFC) reported no significant 5mC CpGs associated with opioid intoxication (13). Another DNAm study in postmortem brain tissue using the same array conducted a co-methylation analysis and identified 6 co-methylated modules related to OUD, involved in response to organic substance and astrocyte and glial differentiation (14). Further, we recently examined neuronal-specific genome-wide 5mC profiles associated with OUD in the OFC, in parallel with 5-hydroxymethylcytosine (5hmC), and identified 397 and 1,740 5mC and 5hmC differential CpGs, respectively (15). We observed epigenetically dysregulated genes implicated in pain signaling and observed enrichment for the Wnt signaling pathway and the G-protein signaling pathway (15). Even though there has been evident progress in the study of DNAm in OUD (or related traits) in recent years, these studies have been limited to examining DNAm at CpG sites using array-based technology.

Compared to array-based technology, single-base resolution sequencing for epigenetic modification detection has increased accuracy, coverage, and resolution in DNAm assessment (16). Bisulfite sequencing has been widely applied in a targeted approach to assessing DNAm of opioid receptors in humans [e.g., child abuse (17), alcohol dependence (18)], and animal models [e.g., pain (19)]. Studies utilizing genome-wide approaches assessing mCpG have been performed in different tissues, including human postmortem brain in the context of schizophrenia (20) and autism (21). However, no previous work has focused on substance use disorders, including OUD.

DNA methylation can also occur in cytosines phosphate-linked to adenine, thymine, and cytosine, known as non-CpG sites (mCpHs). mCpHs is usually low in adult peripheral somatic cells, corresponding to only 0.02% (5). However, a high prevalence of mCpHs is observed in embryonic cells and brain tissue (5). In the brain, mCpHs are highly enriched in neuronal cells, and studies have suggested having a role in gene regulation and brain function (5). Despite mCpHs representing a promising target for the study of brain disorders, little work has focused on evaluating the role of mCpHs in disease etiology. Further, since most DNAm studies have been performed using array-based technology, which only assesses DNAm at CpG sites, very limited work has evaluated mCpH.

In this study, we examined mCpHs at the genome-wide level in the OFC neuronal nuclei of OUD subjects compared with non-OUD subjects. We identified 2,351 differential mCpHs associated with OUD, of which 1,513 were hypomethylated and 838 hypermethylated. Functional annotation analyses showed enrichment for cell-cell communication, glutamatergic synapses, and cholinergic synapses. Gene drug interaction analysis detected 5,690 interactions, including opioid-related drugs as potential drug targets for OUD treatment. Our study highlights the role of mCpH in OUD and reveals important biological pathways and drug targets associated with the disorder.

## Materials and methods

### Sample description

Postmortem human brain OFC from 38 individuals were collected from the brain tissue repository at the National Post-Traumatic Stress Disorder (PTSD) Brain Bank (NPBB) (22), a biorepository from the U.S. Department of Veterans Affairs (VA). Demographics and clinical characteristics are depicted in Table 1.

**TABLE 1:** Demographics of the postmortem OFC specimens obtained from the non-OUD and OUD groups.

	OUD− (*N* = 26)	OUD + (*N* = 12)
**Ancestry**		
African American European American	5	4
	19	8
PMI (μ ± SD)	30.65 ± 8.15	29.6 ± 7.5
Age (μ ± SD)	43.1 ± 11.55	37.6 ± 8.9
Cigarette smoking	13	10
Alcohol dependence	5	3
PTSD	13	12

### Fluorescence-activated nuclei sorting, DNA extraction, and reduced representation oxidative bisulfite sequencing

A total of 100–200 mg of OFC tissue was lysed in homogenization buffer composed of 0.1% Triton, 0.32 M sucrose, 5 mM CaCl2, 3 mM MgCl2, and 10 mM Tris-HCl. After, they were filtered, loaded onto a sucrose cushion, and ultracentrifuged. The nuclei were resuspended in bovine serum albumin with Anti-NeuN-PE being added 4′,6-diamidino-2-phenylindole (DAPI) before sorting. FANS procedure was conducted on a BD 5-laser cell sorting system at the Icahn School of Medicine Flow Cytometry CoRE. The NeuN + nuclei collected (0.5–1 M) were pelleted by centrifugation and processed for DNA extraction according to the DNeasy Blood and Tissue Kit (Cat. #69504, Qiagen) manufacturer’s protocol. Eluted samples were concentrated and stored at −80°C. A total of 400 ng of DNA was used to prepare the methylation library for bisulfite treatment using the NuGEN Ovation RRoxBS Methyl-Seq library preparation kit. The bisulfite treatment converts unmethylated cytosines into uracils. Sequencing was conducted using the Illumina NovaSeq6000 system. Library preparation and sequencing were carried out at the Weill Cornell Epigenomics Core (New York, NY). The complete protocol is described in Rompala et al. (15).

### mCpHs differential analysis

Sequencing reads were mapped to GRCh38, and converted and unconverted cytosines were detected using the Bismark bisulfite read mapper (23). The % of unconverted cytosines was used to represent the beta values. mCpHs were filtered for a minimum of 10x coverage in all samples and further normalized for coverage variability correction. Differential analysis was conducted for single mCpHs using the methylkit R package (24). Age of death, cigarette smoking, ancestry, postmortem interval (PMI), and posttraumatic stress disorder (PTSD) were included as covariates. The false discovery rate (FDR) significance threshold was set as <0.05.

### mCpH annotation

mCpH annotation was performed using genomation (25) and biomaRt (26) R packages. First, annotation of the closest Ensembl transcript ID to the mCpH site was performed using genomation. Briefly, after coercing mCpH sites to GRanges, we used the “annotateWithGeneParts” function to annotate mCpH located at promoters/introns/exons/intergenic regions, followed by the “annotateWithFeatureFlank” function, which performs the annotation of CpG island/shores/flanking regions. Further, the “getAssociationWithTSS” function in genomation was used to calculate the distance to the nearest TSS and annotate the mCpH region with the Ensembl transcript ID. During this step, no threshold was applied for the distance between the mCpH and the annotated gene (TSS distance is provided in Supplementary Table 1). The annotation of gene symbols, descriptions, and biotypes was performed using BiomaRt. By using a known gene dataset from the UCSC genome browser (27), we confirmed the distance between the BiomaRt annotated symbol and the mCpH, where we applied a threshold of 1,500 bp distance. The location of the mCpH was assigned by including 0 = inside the gene, negative number = upstream, and positive number = downstream (Supplementary Table 1). Gene symbols were used for the functional annotation, GWAS enrichment, and drug interaction analysis. For the input for functional annotation, we used two different approaches: All genes and a subset excluding mCpHs in the intergenic regions (i.e., not annotated in the column “Distance <1,500 Up and downstream,” Supplementary Table 1). For GWAS enrichment and drug interaction analysis, we evaluated all annotated genes.

### Functional annotation analysis

Functional annotation was conducted using the enrichR package (28) *via* the web-based tool “Enrichr.” Enrichr performs enrichment analysis with gene sets built based on prior biological knowledge. We selected eight databases, including (1) gene ontology (GO) database, which contains information about gene function describing the process and cellular location (GO_Molecular_Function_2021, GO_Cellular_Component_2021, and GO_Biological_Process_2021), (2) COVID-19 database that includes a collection of genes related to COVID-19 (COVID-19_Related_Gene_Sets_2021), (3) KEGG database that has a collection of known pathways in each of the genes involved (KEGG_2021_Human), (4) Wikipathways, a collaborative platform that maintains a curated collection of biological pathways (WikiPathway_2021_Human), (5) Allen Brain Atlas, an atlas of gene expression integrating it with function and structure (Allen_Brain_Atlas_10x_scRNA_2021), (6) MAGMA database, which contains a collection of gene sets associated with drugs and diseases (MAGMA_Drugs_and_Diseases). Further, we conducted an enrichment analysis using STRING (29), a database of known protein-protein interaction (PPI), which also evaluates databases such as (1) InterPro, which uses protein families and predicted domains to provide the functional annotation, (2) Reactome pathways, a manually curated pathway database, and (3) Tissue expression. We conducted the functional annotation analysis for the whole set of annotated genes (Supplementary Table 2), and for a subset removing sites located in the intergenic regions with a distance greater than 1,500 bp up and downstream using the UCSC software (Supplementary Table 3). Functional annotation for the overlapped annotated genes between mCpHs and 5mC in CpG sites (Supplementary Table 4) was performed using STRING. PPI analysis was performed using the STRING software (29) with the highest confidence score (0.9) and allowing experimentally determined interactions and co-expression. Significant sites were defined as *FDR* < 0.05.

### Cell-type enrichment analysis

Brain cell-type enrichment analysis was carried out using transcriptomic data from human postmortem brain samples to characterize the cell-type enrichment of OUD-associated differential mCpHs in neurons, microglia, astrocytes, oligodendrocytes, and endothelial cell type-specific markers (30).

### Genome-wide association enrichment analysis

Genome-wide association studies enrichment was conducted using FUMA (31). We used the Entrez IDs as input to the GENE2Function web tool. The annotation was performed using the David software (32). The default adjusted *p*-value from FUMA was considered to define significance (adjusted *P* ≤ 0.05). The Bonferroni correction threshold for the *P*-value was set as ≤ 1.89E-4 and described in Supplementary Table 4. We further evaluated the mCpHs annotated with the genes enriched for opioid use-related traits by examining whether the significant mCpH located upstream or downstream of the nearest genes had a known regulatory role. For this, we used JASPAR Transcription Factor Binding Site Database (33) and Encode Database, both linked to the UCSC genome browser web tool (27), that predicts transcription factor binding sites and H3K27Ac marks in the assessed region, respectively.

### Drug interaction analysis

The drug interaction analysis was performed using the Drug Gene Interaction Database (DGIdb) (34) by assessing the interaction between all differential annotated genes and drugs.^1^ Drug interaction was also evaluated for genes involved in synaptic pathways selected from functional annotation using STRING. Cytoscape (35) was used for the visualization of identified drug interactions.

## Results

Neuronal nuclei from postmortem OFC tissue of 38 donors (*n*_OUD_ = 12; *n*_control_ = 26) were analyzed. The samples included males of European and African ancestry (Table 1).

### Differential mCpHs

**FIGURE 1:**
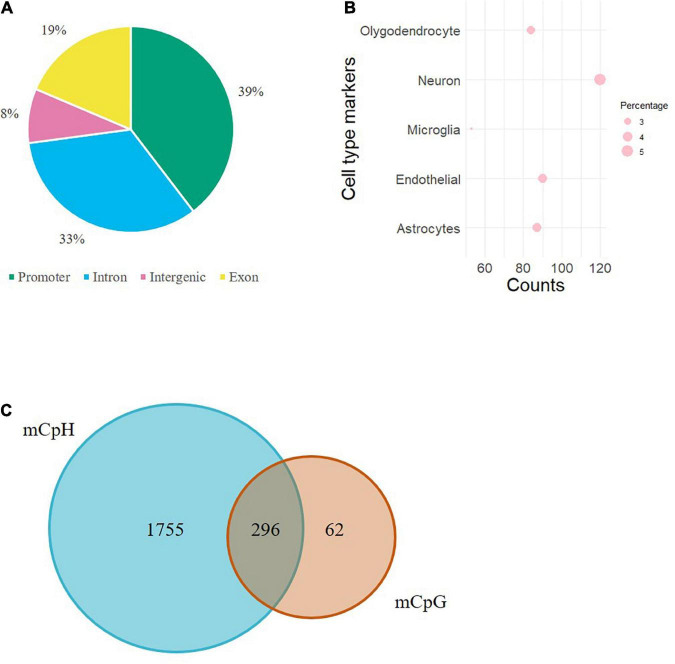
Evaluation of differential mCpHs associated with OUD. **(A)** Genomic location of differential mCpHs. **(B)** Cell-type enrichment of differential mCpHs sites. **(C)** Venn diagram showing the comparison between differential mCpHs and mCpGs.

**FIGURE 2:**
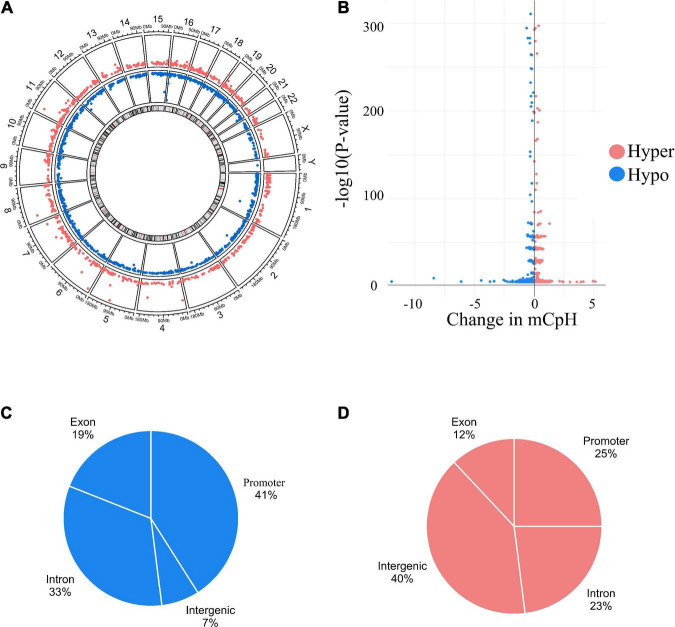
Differential mCpHs sites associated with OUD. **(A)** Circus plot showing hypermethylated and hypomethylated sites. **(B)** Volcano plot of the differential sites. **(C)** Genomic location of hypermethylated and **(D)** hypomethylated differential sites.

Individual mCpHs were evaluated for OUD differential analysis. We observed 2,351 differential mCpHs associated with OUD (Supplementary Table 1) that included 1,513 hypomethylated mCpHs and 838 hypermethylated mCpHs. Assessing the gene features of all differentially methylated CpH loci, most were located in promoter regions (Figure 1A). We compared our differential mCpHs with findings from our previous study focused on 5mC at CpG sites (15) and found that 61 annotated genes overlapped with OUD-associated genes with differential 5mC (Figure 1B and Supplementary Table 4). No significant enrichment was identified for the overlapped genes. Further, cell-type enrichment analysis was conducted to evaluate mCpH sites on gene markers for unique cell types. We observed enrichment of differential mCpH loci on neuron-specific markers, followed by endothelial, astrocytes, oligodendrocytes, and microglia-specific markers (Figure 1C). Differential mCpHs showing hypermethylation were primarily located in the intergenic region, while the hypomethylated mCpHs were more prevalent in promoter regions (Figure 2).

From all the OUD-associated differential mCpHs identified, we were able to detect three annotated genes previously identified in the OUD GWAS literature (36), APBB*2* (*q*-value = 0.007), *CNIH3* (*q*-value = 4.92E-12) and candidate genes (37), the *OPRK1* (*q*-value = 0.020). One of the top associations mapped to the dopamine neurotrophic factor, *CDNF* (*q*-value = 2.96E-25). We also identified genes previously found in epigenome-wide association studies (EWAS) of opioid dependence (6), including *RERE* (*q*-value = 4.79E-53), and *CFAP77* (*q*-value = 3.91E-25).

### Functional annotation

**FIGURE 3:**
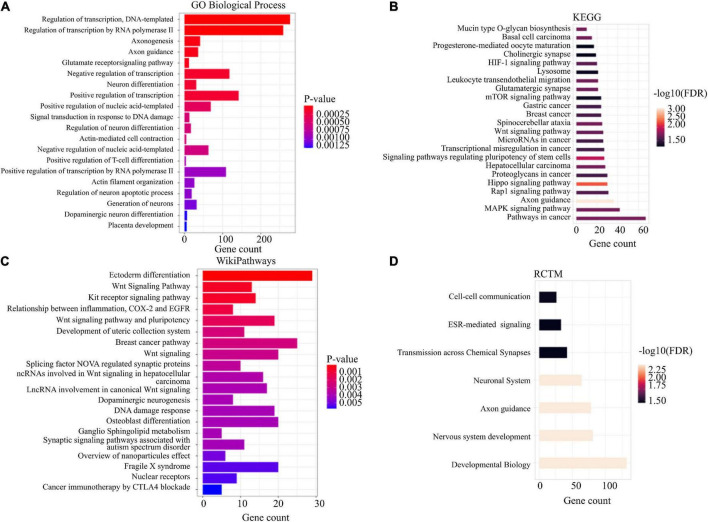
Functional enrichment. Enrichment analysis was conducted using EnrichR **(A,B)** and STRING software **(C,D)** for GO biological process, wikiPathways, KEGG, and RCTM.

Functional annotation was performed using enrichR (28) and STRING (29) for all annotated genes and for a subset excluding intergenic sites. When we evaluated all annotated genes using enrichR, we observed an enrichment for glutamate receptor signaling pathway (*p*-value = 5.67E-05), regulation of neuron differentiation (*p*-value = 5.27E-04), and dopaminergic neuron differentiation (*p*-value = 1.24E-03) (Figure 3A). We also detected enrichment of differential mCpHs in Relationship between inflammation, COX-2 and EGFR (*p*-value = 1.10E-03; Figure 3B), Wnt Signaling pathway (*p*-value = 5.20E-04; Figure 3B), and dopaminergic neurogenesis (*p*-value = 3.97E-03; Figure 3B). Using STRING, we observed enrichment for the cholinergic synapse (*FDR* = 0.047; Figure 3C), glutamatergic synapse (*FDR* = 0.021; Figure 3C), cell-cell communication (*FDR* = 0.034; Figure 3D); and transmission across chemical synapses (*FDR* = 0.036; Figure 3D). Additional functional annotation results are included in Supplementary Table 2.

Although we observed changes in the enrichment analysis when evaluating the subset excluding the intergenic sites, we also observed some overlapping enrichment, including glutamate receptor signaling pathway (*p*-value = 4.30E-05), regulation of neuron differentiation (*p*-value = 0.0016), dopaminergic neuron differentiation (*p*-value = 0.0024), Relationship between inflammation, COX-2 and EGFR (*p*-value = 0.0079), Wnt Signaling pathway (*p*-value = 0.00028), dopaminergic neurogenesis (*p*-value = 0.0049), cholinergic synapse (*FDR* = 0.022), glutamatergic synapse (*FDR* = 0.0084) (Supplementary Table 3). Comparing all annotated genes and the subset, we observed an overlap of enrichment terms of 66 (Allen Brain Atlas), 67 (COVID-19 related genes), 214 (GO biological terms), 20 (GO cellular component), 54 (Go molecular function), 44 (KEGG), 29 (MAGMA drugs and diseases), and 54 (WikiPathways) using enrichR, and 9 (Tissues), 1 (RCTM), 1 (PFAM), 19 (KEGG), 3 (Interpro), 3 (Diseases) using STRING.

### Protein-protein interaction analysis

**FIGURE 4:**
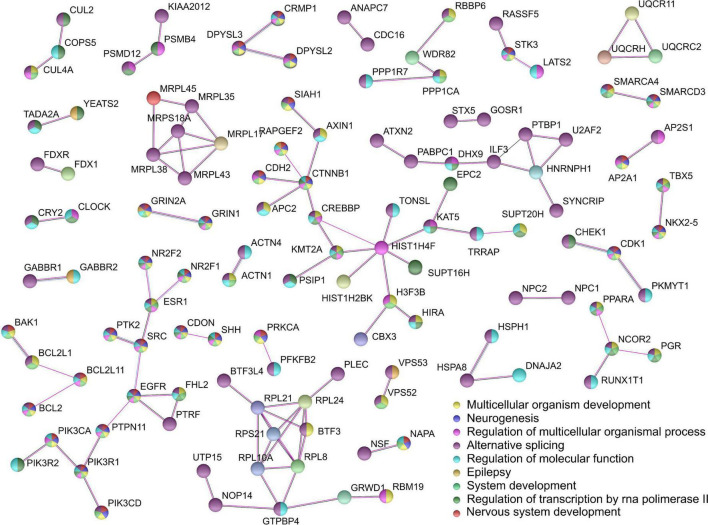
Protein-protein interaction network analysis. We used STRING selecting experiments and co-expression with the highest confidence value (0.9).

The PPI analysis of all annotated genes from the OUD-associated differential mCpHs was performed using STRING (29) (Figure 4). We chose nine significantly enriched pathways, including nervous system development (*FDR* = 4.76e-20), multicellular organism development (*FDR* = 1.04e-18), neurogenesis (*FDR* = 1.04e-18), system development (*FDR* = 5.26e-18), alternative splicing (*FDR* = 2.18e-14), regulation of multicellular organismal process (*FDR* = 8.62e-12), regulation of molecular function (*FDR* = 2.79e-09), regulation of transcription by RNA polymerase II (*FDR* = 1.81e-09), and epilepsy (*FDR* = 0.0388). Three interactions were associated with cholinergic synapses (BAK1-BCL2L1-BCL2L11-BCL2*, NR2F2-ESR1-SRC-EGFR-PTPN11-PIK3R1*-PIK3CD*-PIK3CA*-*PIK3* R2*, PRKCA*-*PFKFB2*), and two with glutamatergic synapses (GRIN2A*-GRIN1*, PRKCA*-*PFKFB2*), where * indicates the genes related with synaptic pathways.

### Genome-wide association studies enrichment of differential mCpHs

Genome-wide association studies enrichment analysis (Table 2 and Supplementary Table 5) of all annotated genes from the OUD-associated differential mCpHs identified enrichment for two opioid gene sets—Methadone dose in opioid dependence (*p*-value = 3.00E-03; *SORCS1, GRK5, E2F7, SPRY2, TRIB2*, and *BCL11A*), and Opioid dependence (*p*-value = 6.18E-03; *CNIH3, TACC2*). In addition, we observed enrichment for Cannabis use (*p*-value = 8.83E-09), Cognitive decline rate in late mild cognitive impairment (*p*-value = 4.40E-07), Neuroticism (*p*-value = 6.03E-05), and Smoking initiation (*p*-value = 8.11E-05).

**TABLE 2:** Genome-wide association studies enrichment analysis of annotated genes from the OUD-associated differential mCpHs.

GeneSet	*N*	*n*	*P*-value	Adjusted *p*
Cannabis use	10	7	8.83E-09	1.99E-06
Schizophrenia	827	60	3.52E-07	5.82E-05
Cognitive decline rate in late mild cognitive impairment	122	18	4.40E-07	6.65E-05
Cognitive ability, years of educational attainment or schizophrenia (pleiotropy)	197	23	8.86E-07	1.24E-04
Intelligence (MTAG)	313	30	1.51E-06	1.83E-04
Autism spectrum disorder, attention deficit-hyperactivity disorder, bipolar disorder, major depressive disorder, and schizophrenia (combined)	46	10	4.69E-06	4.06E-04
General cognitive ability	256	24	2.34E-05	1.34E-03
Systolic blood pressure x smoking status (current vs. non-current) interaction (2 df test)	111	14	5.01E-05	2.27E-03
Neuroticism	99	13	6.03E-05	2.61E-03
Smoking initiation	51	9	8.11E-05	3.22E-03
Diastolic blood pressure x smoking status (current vs. non-current) interaction (2 df test)	107	13	1.35E-04	4.72E-03
Cognitive performance	84	11	2.24E-04	6.36E-03
Bitter alcoholic beverage consumption	91	11	4.54E-04	1.14E-02
Feeling tense	19	5	4.72E-04	1.14E-02
Systolic blood pressure x smoking status (ever vs. never) interaction (2 df test)	95	11	6.57E-04	1.47E-02
Major depressive disorder	210	18	6.90E-04	1.49E-02
Neuroticism	149	14	1.09E-03	2.04E-02
General factor of neuroticism	104	11	1.40E-03	2.37E-02
Bipolar I disorder	90	10	1.56E-03	2.54E-02
Smoking status (ever vs. never smokers)	190	16	1.60E-03	2.57E-02
Methadone dose in opioid dependence	40	6	3.00E-03	3.97E-02
Alcohol dependence	54	7	3.24E-03	4.14E-02
Bipolar disorder	656	38	3.39E-03	4.21E-02
Cognitive function	85	9	3.67E-03	4.37E-02
Feeling fed-up	30	5	4.16E-03	4.72E-02
Cognitive function	85	11	1.80E-03	1.94E-02
Experiencing mood swings	40	7	2.15E-03	2.25E-02
Bipolar I disorder	90	11	2.86E-03	2.76E-02
Nicotine dependence	24	5	4.23E-03	3.49E-02
Cognitive ability	46	7	4.85E-03	3.76E-02
Feeling hurt	25	5	5.09E-03	3.80E-02
Bipolar disorder with mood-incongruent psychosis	16	4	5.26E-03	3.88E-02
Feeling worry	48	7	6.16E-03	4.42E-02
Opioid dependence	3	2	6.18E-03	4.42E-02
Pulse pressure x alcohol consumption (light vs. heavy) interaction (2 df test)	17	4	6.63E-03	4.70E-02
HDL cholesterol levels x alcohol consumption (regular vs. non-regular drinkers) interaction (2 df)	129	13	6.73E-03	4.72E-02

Significant enrichment GeneSet involved in drug use, psychiatric disorders, and cognitive function. *N* means the number of genes in the GeneSet and *n* represents the number of genes detected in the input.

### Gene-drug interaction analysis

**FIGURE 5:**
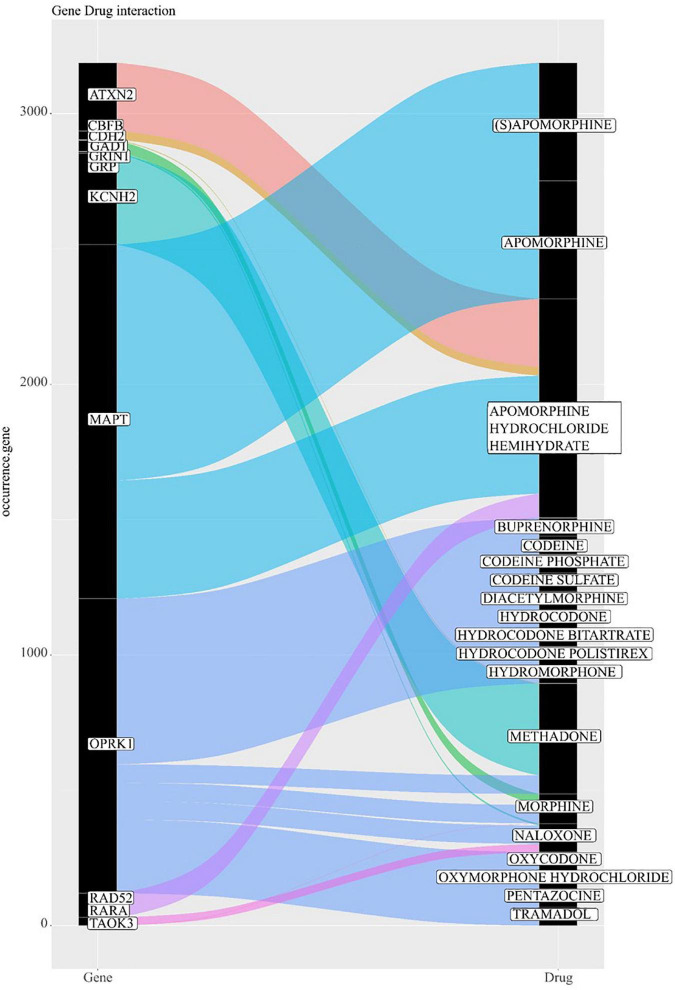
Gene-drug interactions with opioids. The figure shows all genes that interact with 15 selected opioids. OPRK1 interacts with most opioid-related drugs.

Drug interaction analysis showed 3,420 interactions (Supplementary Table 6) between the annotated genes with differential mCpHs and drugs. We evaluated the interactions with 15 opioids: Apomorphine, codeine, diacetylmorphine, hydrocodone, methadone, morphine, oxycodone, oxymorphone, tramadol, methadone, propoxyphene, fentanyl, hydromorphone, heroin, levorphanol, meptazinol, naloxone, pethidine, buprenorphine, and pentazocine. As a result, we found 12 annotated genes in our differential mCpHs that interacts with opioids (Figure 5), *ATXN2, CBFB, CDH2, GAD1, GRIN1, KCNH2, MAPT, OPRK1, RAD52, RARA, GRP*, and *TAOK3*. We also identified genes with known interactions with pergolide mesylate (*KCNH2*), lofexidine (*ADRA2B*), and tizanidine (*ADRA2B*). Finally, we evaluated drug-gene interactions for genes associated with two specific pathways detected in the functional annotation analysis: glutamatergic synapses (KEGG, Supplementary Figure 2), and cholinergic synapses (KEGG, Supplementary Figure 3). We further evaluated the location of differential mCpH in the genes with described interaction with opioids, and observed that *ATXN2, CBFB, CDH2, GRIN1, KCNH2, OPRK1, RARA*, and *GRP* (one mCpH in each gene), *MAPT* and *RAD52* (two mCpHs each) were located inside the gene, *GAD1* and *GRP* (one mCpH) were located upstream, and *TAOK3* was annotated in an intergenic region.

## Discussion

Here, we examined the role of non-CpG methylation in OUD, which to date had not been investigated. Evaluating genome-wide differential mCpHs associated with OUD in human postmortem OFC tissue, revealed 2,351 differential mCpHs enriched for glutamatergic synapses and cholinergic synapses. Gene-drug interaction analysis showed ten annotated genes with differential mCpHs interacting with opioid-related drugs, including lofexidine and tizanidine, both currently used for OUD treatment. These results highlight neuronal mCpH as an important regulatory mechanism in the pathophysiology of OUD.

Interestingly, several of the differential mCpHs mapped to genes previously described in OUD GWAS studies: *OPRK1, APBB2*, and *CNIH3*. *OPRK1* encodes a kappa opioid receptor and is known to be associated with morphine (38) and alcohol dependence (39). In our analysis, this gene was hypomethylated and showed interactions with several opiates. A recent study reported a role of *OPRK1* genetic variants in pain modulation (40). Further, a previous candidate gene study found associations of *OPRK1* variants with methadone dosage in a Chinese population (*n* = 801) (41). *APBB2* overexpression is associated with β-amyloid (42) and has been associated with Alzheimer’s disease (43). GWAS studies have suggested an association of *APBB2* (*p*-value = 1.8 × 10^–9^) with OUD in African Americans (AA) of two independent samples of the SAGE cohort (*n* = 3,318 and *n* = 1,311) (44). More recently, variants at *APBB2* were also associated amphetamine use in Taiwanese individuals with OUD receiving methadone treatment (*n* = 344) (45). *CNIH3* is a glutamate receptor auxiliary protein that regulates trafficking properties in glutamate receptors. This gene has been associated with daily opioid use in an Australian cohort (*n* = 1,167), a finding replicated in the Yale Penn cohort (*n*_daily_ = 643; *n*_lifetime_ = 157) (46).

We also identified mCpHs in other genes enriched for opioid-related gene sets: *SORCS1, GRK5, E2F7, SPRY2, TRIB2, BCL11A, CNIH3*, and *TACC2*. *SORCS1* is a regulator of the synaptic trafficking required for glutamatergic synaptic transmission in mice brain (47) being associated with Alzheimer’s disease risk in a candidate gene study (48). *GRK5* is a kinase that phosphorylates G-protein-coupled receptors, including dopamine and opioid receptors. An up-regulation of *GRK5* has been described during initial morphine treatment in male Sprague–Dawlay rats and suggested to play a role in the regulation of opioid signaling (49). Genetic variants at the *GRK5* gene have been associated with the regulation in methadone dosage in heroin dependence (50). *SPRY2* is an intracellular inhibitor of growth factors involved in stress sensitivity (51). *TRIB2* is a member of the tribble family; risk alleles mapping to this gene have been also associated with methadone dose in an African Americans population (52). *BCL11A* is a zinc-finger protein, in which its deletion is associated with higher levels of fetal hemoglobin (HbF). Although we did not observe expression changes of HbF, we did observe a differential gene expression in the HBB associated with OUD in our previous study (15).

Here, we also identified an OUD-associated differential mCpH site near the dopamine neurotrophic factor *CDNF*, involved in dopaminergic neuron differentiation and shown to have neuroprotective and neurorestorative effects in these neurons (53). Further, two genes with differential mCpHs in OUD, *RERE*, and *CFAP77*, were previously identified in an epigenome-wide association study from our group evaluating differential 5mC associated with opioid dependence in whole blood samples from European–American women (*n* = 220) (6). *RERE* is highly expressed in the brain and plays a role in brain development, retinoic acid signaling, and gene regulation (54). *CFAP77* is involved in cell projection. Despite the unclear role of these two genes in OUD, the replication of these epigenetic markers across tissues suggests a promising important role in the pathophysiology of OUD.

Functional annotations of the differential mCpHs using KEGG highlighted two enriched pathways—glutamatergic and cholinergic synapses. The glutamatergic system has been implicated in opioid-induced hyperalgesia, where patients undergoing chronic opioid therapy endure acute pain and tolerance (55). In addition, studies have suggested that morphine exposure may decrease glutamate transporter expression (56). There is also evidence that glutamate transporters interact with opioid receptors (56). The link between glutamatergic signaling and opioid addiction could be also due to its role in learning and memory formation, where glutamatergic circuits may be recruited to create and maintain memories of reward, aversion, and reinforcement processes (57). The cholinergic system, implicated in several brain functions, including sensory processing, sleep, memory, learning, and attention (58, 59), has been suggested as a promising target for OUD treatment (60). Furthermore, opioids modulate cholinesterase activity, an enzyme involved in regulating cholinergic response (61). Moreover, we observed enrichment of the Wnt signaling pathway, previously involved in opioid-related withdrawal in mice (62). Our results show that dysregulated mCpHs sites target genes involved in both cholinergic and glutamatergic systems as well as Wnt signaling, all previously implicated in opioid-related traits.

We also observed several OUD-associated differential mCpHs genes involved in alternative splicing as predicted with the Protein-Protein Interaction analysis. A recent study assessing the interaction of alternative splicing in opioid-induced hyperalgesia in the nucleus accumbens (NAc) and trigeminal ganglia of mice identified four genes with more than four nodes connections in the NAc: *Grin1, Src, Dnm1*, and *Pxn* (63), three of which were detected in our analysis with differential mCpHs, *GRIN1, SRC*, and *DNM1*. The *GRIN1* gene was also identified with known interactions with opiates. A study evaluating mRNA alternative splicing in the human postmortem dlPFC, NAc, and midbrain associated with OUD identified eight differential spliced genes in morphine addiction (64), one of which was identified in our analysis: *GABBR1*. In addition, they observed five spliced genes in the dlPFC, NAc, and ventral midbrain (64), including two genes with differential mCpHs found in our work: *HERC1* and *BIN1*. These results support an important gene regulatory role of OUD-linked differential mCpHs.

Genome-wide association studies enrichment of annotated genes showing differential mCpHs showed associations with opioid dependence as well as other substance use such as tobacco, alcohol, and cannabis. Tobacco smoking is considered a major risk factor for OUD development (65). Further, studies have shown co-morbidities between OUD and other substance use disorders, including cannabis (66), nicotine (67), and alcohol (66, 68). We also observed enrichment for cognitive decline and impairment. Cognitive deficit is one of the withdrawal symptoms of OUD (69). Our findings suggest that epigenetic mechanisms may underlie the co-occurrence of SUDs. However, studies in larger and better-powered samples may help disentangle potential shared epigenetic mechanisms in SUDs comorbidities.

Gene-drug interaction analysis showed interactions of annotated differential mCpH genes with opioid-related drugs, including lofexidine (helps with physical symptoms of opioid withdrawal), tizanidine (used to treat withdrawal symptoms in heroin users), and pergolide mesylate (used to treat cocaine dependence). The genes interacting with opioids included *GRIN1* and *OPRK1*, described above. *GAD1* is a glutamic acid decarboxylase related to glutamate-dependent acid resistance; candidate gene studies reported genetic variants associated with heroin addiction (70). *KCNH2*, a Potassium Voltage-Gated Channel Subfamily H Member 2, component of potassium channels, was also detected in our study associated with pergolide mesylate, a drug used to treat cocaine dependence (71). Further, *KCNH2* polymorphisms were shown as a potential risk factor associated with QT prolongation (interval between the ventricle depolarization and repolarization) under low doses of methadone treatment in a French cohort (72). We also observed the interaction between *ADRA2B* and tizanidine, lofexidine, and dopamine. *ADRA2B* is involved in the thermoregulatory effects induced by the opiate meperidine in mice (73). Interestingly, both tizanidine (74) and lofexidine has been used to treat symptoms related to opioid withdrawal (75).

Comparing the non-CpG 5mC results with CpG 5mC described in Rompala et al. (15), we observed more OUD-associated differential changes in mCpHs than mCpGs. Further, mCpHs enrichment showed a higher number of significantly enriched pathways, including KEGG signaling pathways, glutamatergic and cholinergic synapses. Drug interaction analysis also showed a higher number of annotated genes with differential mCpHs than mCpGs associated with opioids, suggesting that 5mC at non-CpG sites in OFC neurons may be more functionally relevant than 5mC at CpG sites in the context of OUD. Our results highlight the importance of studying 5mC at non-CpG sites. We also evaluated the differential gene expression in association with OUD in OFC previously described in Rompala et al. (15) on the same cohort as in the current study; however, no significant OUD-associated differential expression was observed of the genes identified here with differential mCpH (*p*-value range = 0.091–0.987; Supplementary Table 7).

We further evaluated whether the identified differential mCpHs would be targeted for activation of gene expression through the prediction of transcriptional factor binding site (TFBS) and enrichment of H3K27Ac markers (Table 3). We observed that seven of the differential mCpHs identified here that was located upstream of the annotated gene showed predicted TFBS. A total of five mCpHs were located in exon, of which four have predicted TFBS. A total of eight were detected inside introns, of which five showed predicted TFBS sites. The only identified differential mCpH in the intergenic region was also detected with predicted TFBS. Further, 14 of these mCpH sites were observed with H3K27Ac marker, suggesting a potential role in gene regulation (Table 3).

**TABLE 3:** Detailed information about transcription factor binding site (TBFS) and H3K27Ac mark.

Chr	Start	Strand	Gene	Gene location	Predicted TFBS	H3K27Ac mark
chr4	41214810	–	*APBB2*	Upstream	*ZNF701*	Yes
chr1	224434745	+	*CNIH3*	Exon 1	*ZNF610*	Yes
chr10	14825089	–	*CDNF*	Intron 3	–	–
chr1	8817779	+	*RERE*	Upstream	*ZIC1, ZIC4, ZIC5, CTCFL, CREB3L4*	Yes
chr9	132410191	+	*CFAP77*	Exon 1	–	Yes
chr10	107131629	–	*SORCS1*	Intron 1	*SORCS1*	–
chr10	119204183	+	*GRK5*	Upstream	*ESRRA*	–
chr12	77066065	–	*E2F7*	Upstream	*ZNF343*	Yes
chr13	80343091	–	*SPRY2*	Upstream	*ZNF816*	Yes
chr2	12717287	–	*TRIB2*	Exon 1	*ZNF530*	–
chr2	60554096	+	*BCL11A*	Exon 1	*ZNF343* and *E2F6*	Yes
chr10	122239856	+	*CNIH3*	Intron 18	*PRDM15*	–
chr10	122233129	–	*TACC2*	Intron 16	–	Yes
chr9	137139932	+	*GRIN1*	Intron 1	–	Yes
chr20	37384438	+	*SRC*	Intron 5	*WT1, SP9, ZNF135, RPBJL, ZEB1, PLAG1, PLAGL1*	Yes
chr9	128216039	+	*DNM1*	Intron 1	*RREB1, MAZ, KLF4, ZNF281, ZNF148, KLF5, ZNF263, PATZ1, KLF1, SP2, SP4, SP5*	–
chr6	29633436	–	*GABBR1*	Upstream	*NFIC*	Yes
chr15	63833922	+	*HERC1*	Exon 1	*MAF, MAFA, ZNF93, FERD3L*	Yes
chr2	127107493	–	*BIN1*	Upstream	*ZNF610*	Yes
chr7	150974535	+	*KCNH2*	Intron 2	*MAZ, ZNF148, SP5, WT1*	Yes
chr2	96084211	–	*ADRA2B*	Intergenic	*CTCFL, TFAP2A, TFAP2B, TFAP2C, PPARA*:*RXRA*	–

The table includes information about TFBS and H3K27Ac mark for the sites discussed.

This study has limitations relevant to interpreting the data. We studied a small sample of subjects that included only 12 individuals diagnosed with OUD, of which we only evaluated differences in methylation levels of males, most of European ancestry. Furthermore, the potential effects of comorbidities often associated with OUD and other SUDs were not fully addressed due to lack of power due to the limited sample size. Future work aims to increase the sample size and evaluate the effects of ancestry, sex, as well as the influence of comorbidities in a better-powered, more diverse cohort.

## Conclusion

In conclusion, this is the first examination of non-CpG methylation in OUD, identifying 2,351 differential mCpHs that included well-known opioid-related genes, such as the *OPRK1* gene. We identified cholinergic and glutamatergic synapses among the functional pathways associated with the differential mCpHs. Drug interaction analysis also showed several genes interacting with opioids, including the *OPRK1, GRIN1*, and *KCNH2*, as well as gene interactions with drugs used for the treatment of opioid-related withdrawal symptoms. Our findings reveal mCpHs as a crucial regulatory mechanism in OUD and may help shed light on future therapeutic and preventive strategies for treating this disorder.

## Members of the Traumatic Stress Brain Research Group

Victor E. Alvarez, David Benedek, Alicia Che, Dianne A. Cruz, David A. Davis, Matthew J. Girgenti, Ellen Hoffman, Paul E. Holtzheimer, Bertrand R. Huber, Alfred Kaye, John H. Krystal, Adam T. Labadorf, Terence M. Keane, Mark W. Logue, Ann McKee, Brian Marx, Deborah Mash, Mark W. Miller, Crystal Noller, JM-O, William K. Scott, Paula Schnurr, Thor Stein, Robert Ursano, Douglas E. Williamson, Erika J. Wolf, and Keith A. Young.

## Data availability statement

The original contributions presented in this study are included in the article/Supplementary material, further inquiries can be directed to the corresponding author.

## Ethics statement

The studies involving human participants were reviewed and approved by the IRB Committee from the Department of Veteran’s Affairs. The patients/participants provided their written informed consent to participate in this study.

## Author contributions

SN was responsible for data analysis, interpretation, and manuscript writing. GR and DN-R contributed to the data analyses. YH contributed to data generation and study design. TSBRG contributed to data collection. JM-O contributed to data generation and interpretation, study design, and supervision of manuscript writing. All authors contributed to the revision of the manuscript and approved its final version.

## Footnotes

1
https://www.dgidb.org
