# Supplementary material for: CpH methylome analysis in human cortical neurons identifies novel gene pathways and drug targets for opioid use disorder
Source: Front Psychiatry. 2023 Jan 19;13:1078894. doi: 10.3389/fpsyt.2022.1078894 (PMC9892724; doi:10.3389/fpsyt.2022.1078894)
Supplement: Supplementary file 1 [file Data_Sheet_1.PDF]

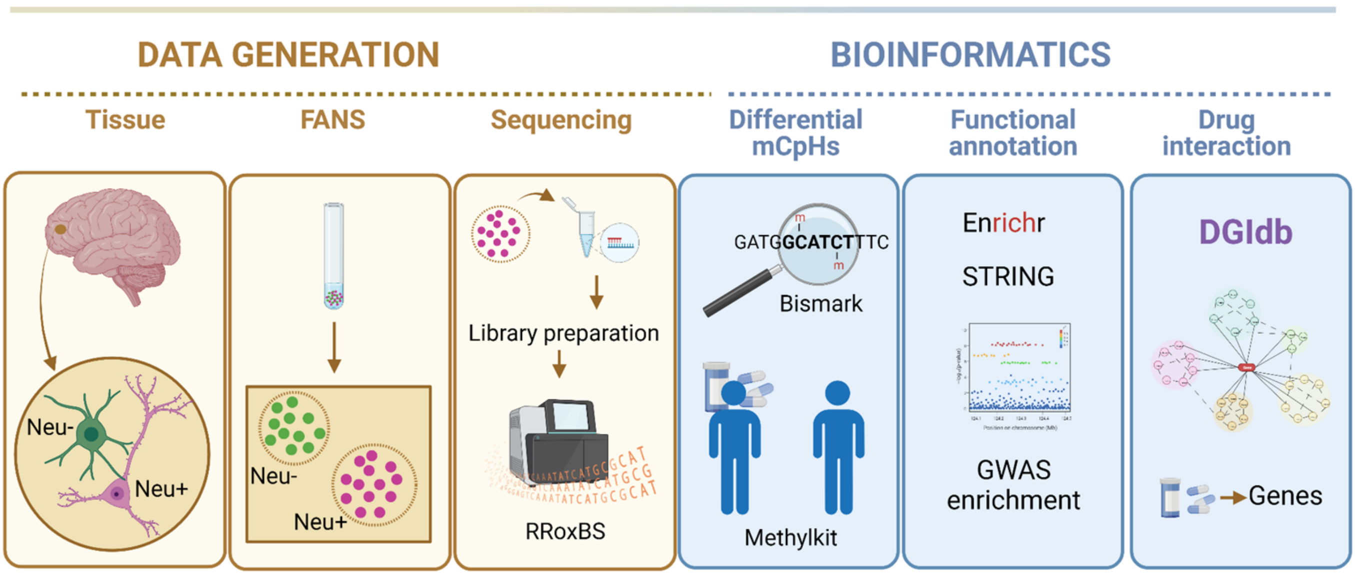

Supplementary Figure 1. Study workflow.

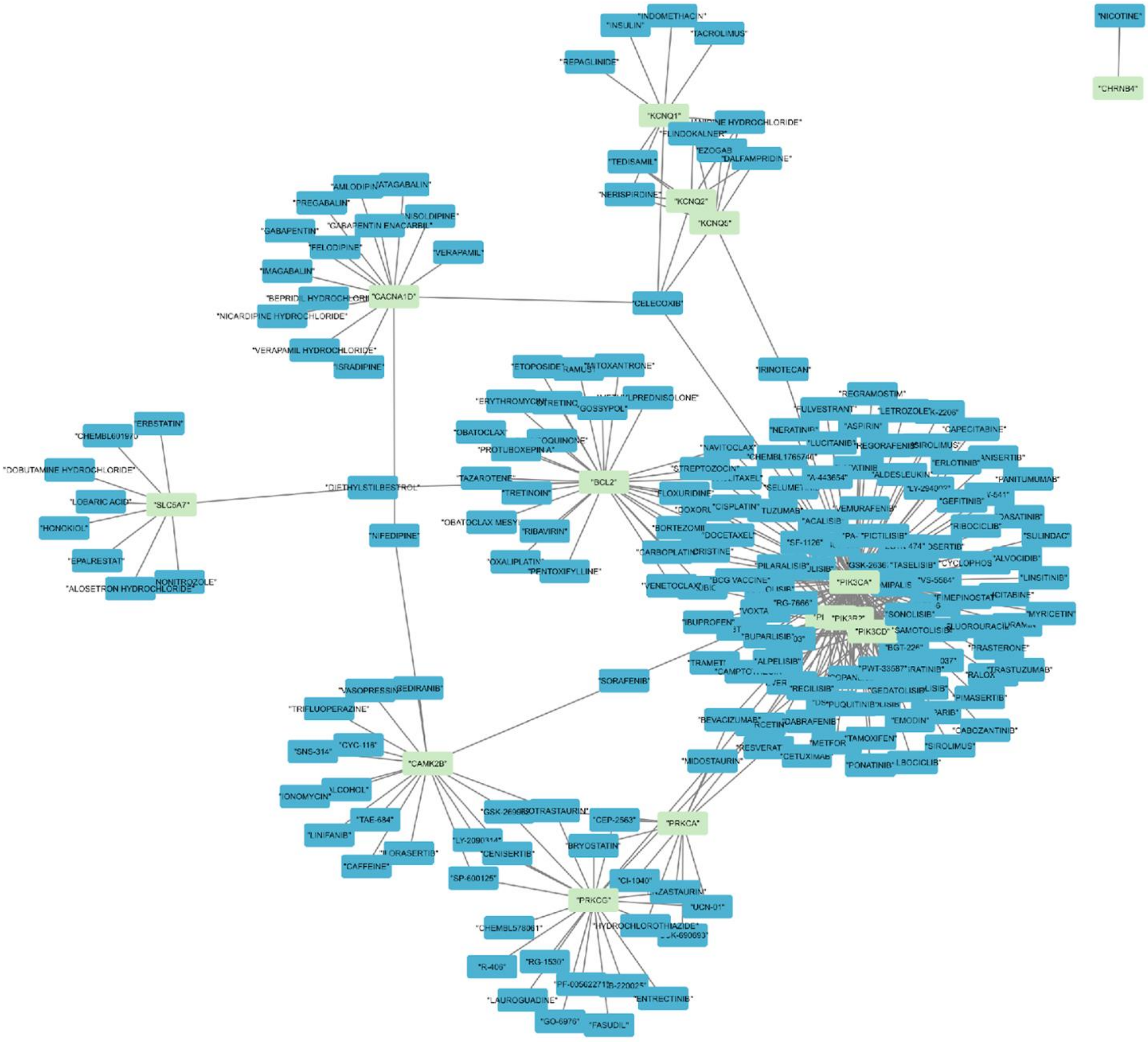

Supplementary Figure 2. Exploratory analyses for gene-drug interactions involved in cholinergic synapses. The network shows genes (green) involved in the cholinergic synapses and drugs interacting with them (blue).

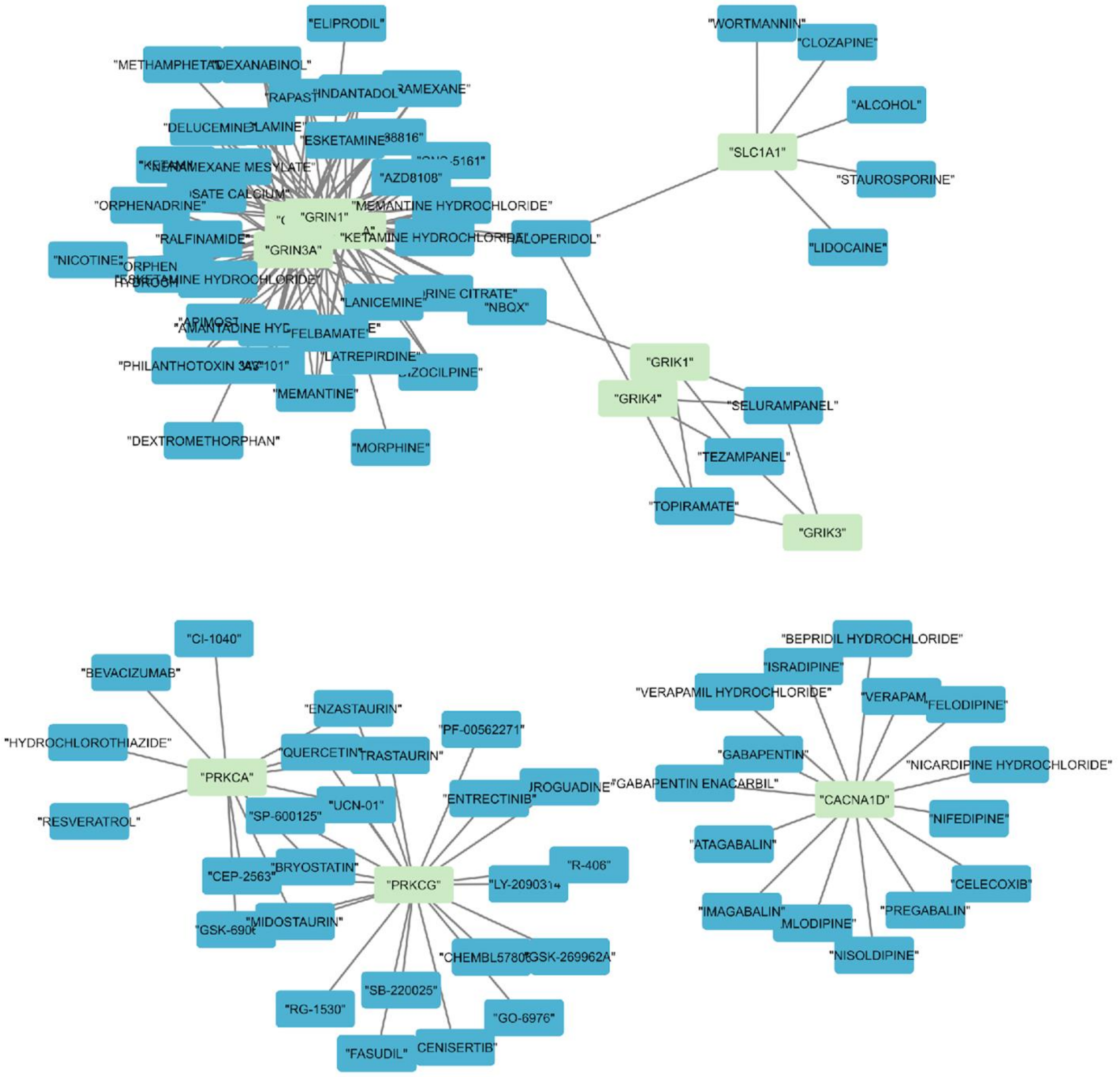

Supplementary Figure 3. Exploratory analyses for gene-drug interactions involved in glutamatergic synapses. The network shows in green the genes involved in glutamatergic synapses and in blue the drugs with described interactions.
